# Metabolomic Profiling of Aqueous Humor from Pathological Myopia Patients with Choroidal Neovascularization

**DOI:** 10.3390/metabo13080900

**Published:** 2023-07-30

**Authors:** Qiaoling Wei, Zhiqiang Yu, Xianjin Zhou, Ruowen Gong, Rui Jiang, Gezhi Xu, Wei Liu

**Affiliations:** 1 Department of Ophthalmology, Eye and ENT Hospital, Shanghai Medical College, Fudan University, Shanghai 200031, China; 2 Shanghai Key Laboratory of Visual Impairment and Restoration, Fudan University, Shanghai 200031, China; 3 NHC Key Laboratory of Myopia (Fudan University), Key Laboratory of Myopia, Chinese Academy of Medical Sciences, Shanghai 200031, China; 4 Ocular Trauma Center, Eye and ENT Hospital, Shanghai Medical College, Fudan University, Shanghai 200031, China

**Keywords:** choroidal neovascularization, pathological myopia, metabolomics, aqueous humor, carbohydrate metabolism, glycometabolism

## Abstract

Choroidal neovascularization (CNV) is a severe complication observed in individuals with pathological myopia (PM). Our hypothesis is that specific metabolic alterations occur during the development of CNV in patients with PM. To investigate this, an untargeted metabolomics analysis was conducted using gas chromatography–mass spectrometry (GC–MS) and liquid chromatography–mass spectrometry (LC–MS) on aqueous humor (AH) samples obtained from meticulously matched PM patients, including those with CNV (n = 11) and without CNV (n = 11). The analysis aimed to identify differentially expressed metabolites between the two groups. Furthermore, the discriminative ability of each metabolite was evaluated using the area under the receiver operating characteristic curve (AUC). Enriched metabolic pathways were determined using the KEGG and MetaboAnalyst databases. Our results revealed the detection of 272 metabolites using GC–MS and 1457 metabolites using LC–MS in AH samples. Among them, 97 metabolites exhibited significant differential expression between the CNV and non-CNV groups. Noteworthy candidates, including D-citramalic acid, biphenyl, and isoleucylproline, demonstrated high AUC values ranging from 0.801 to 1, indicating their potential as disease biomarkers. Additionally, all three metabolites showed a strong association with retinal cystoid edema in CNV patients. Furthermore, the study identified 12 altered metabolic pathways, with five of them related to carbohydrate metabolism, suggesting their involvement in the occurrence of myopic CNV. These findings provide possible disease-specific biomarkers of CNV in PM and suggest the role of disturbed carbohydrate metabolism in its pathogenesis. Larger studies are needed to validate these findings.

## 1. Introduction

Pathological myopia (PM) represents a prevalent cause of visual impairment and blindness on a global scale. Previous investigations have demonstrated a prevalence of PM-associated visual impairment ranging from 0.1% to 1.4% [1]. A significant subset of PM patients experiences the development of myopic choroidal neovascularization (CNV), a condition frequently leading to abrupt central vision loss. If left untreated, CNV lesions can result in scarring, leading to the expansion of macular atrophy and irreversible visual impairment. Despite the fact that only a fraction of PM patients (approximately 5 to 11%) progresses to CNV, the resultant visual loss places a substantial emotional and economic burden on young individuals with myopia [1,2,3,4]. Hence, elucidating the underlying mechanisms and identifying disease-specific biomarkers associated with myopic CNV is of utmost importance in developing effective preventive strategies and treatments. However, the precise relationship between PM progression and the risk of developing myopic CNV remains elusive.

Metabolomics has emerged as a promising biochemical technique for identifying disease-specific molecules and elucidating altered metabolic pathways associated with diseases, enabling metabolic profiling of healthy individuals [5]. In the context of ocular diseases, including cataracts, Fuchs’ syndrome, myopia, and age-related macular degeneration (AMD), metabolomic analysis has been extensively employed to detect the metabolic characteristics [6,7,8,9,10,11,12,13]. Aqueous humor (AH), as a readily accessible and safe sample, has been widely used to investigate intraocular microenvironmental changes. While metabolomics holds the potential to unveil metabolic signatures during CNV formation, to our knowledge, its application to AH samples from myopic CNV patients remains unexplored, thereby presenting an opportunity to gain more precise insights into intraocular microenvironmental changes.

In this study, our hypothesis is that performing untargeted metabolomics analysis on AH samples obtained from carefully matched PM patients with and without CNV will reveal disease-specific metabolic alterations during CNV development. In the current study, owe employed both liquid chromatography–mass spectrometry (LC–MS) and gas chromatography–mass spectrometry (GC–MS) to enhance the coverage of the metabolites. Our objectives were to identify metabolites and possible altered metabolic pathways associated with CNV formation in individuals with PM and to quantify their performance in detecting this severe complication.

## 2. Materials and Methods

### 2.1. Study Participants

We conducted a cross-sectional, observational study involving consecutive PM patients who underwent intraocular injection for myopic CNV or implantable collamer lens (ICL) V4c implantation for optic correction at the Eye Department of the Eye & ENT Hospital of Fudan University, Shanghai, China, between February 2021 and July 2022. An approval from the Ethics Committee of the Institutional Review Board of the EYE & ENT Hospital was obtained (no. 2023-YS-016), and all recruited patients provided informed consent for the publication of this paper. The study adhered to the ethical principles outlined in the Declaration of Helsinki.

PM was defined as a spherical equivalent refractive error (SER) less negative than –6.0 D or an axial length (AL) greater than 26.5 mm, along with typical PM-related chorioretinal degeneration fundus. All patients (n = 11) enrolled in the CNV group were initially diagnosed with active PM-related CNV and had not undergone any previous treatments. The confirmation of active CNV status was based on a thorough assessment that included fundus examination, optical coherence tomography (OCT), and optical coherence tomography angiography (OCTA). The PM control group (n = 11) consisted of individuals who underwent implantation of ICL V4c for optical correction. These individuals were carefully selected as they showed no evidence of CNV and had not undergone any previous therapeutic interventions for CNV. To ensure rigorous comparability, both groups were meticulously matched in terms of age, sex, and AL. Patients with cataracts, a history of eye surgeries or uveitis, and systemic diseases such as diabetes and hypertension were excluded. The clinical characteristics of the patients, including fundus status, CNV sizes, subretinal fluid, and retinal cystoid edema, were recorded.

### 2.2. Sample Collection and Metabolomics Analysis

Approximately 0.1–0.15 mL of AH was obtained from each patient through anterior chamber paracentesis using a 1 mL needle before surgery or intraocular injection treatment. The AH samples were immediately placed into sterile tubes and stored at −80 °C until further investigation.

Metabolomic analysis was performed using gas chromatography–mass spectrometry (GC–MS) and liquid chromatography–mass spectrometry (LC–MS) following established protocols [12,13,14]. Quality control samples were prepared by pooling equal volumes of each AH sample to assess the stability and reproducibility of the untargeted metabolic analysis. The current untargeted metabolic analysis was performed via GC–MS and LC–MS. For GC analysis, AH samples were analyzed on an Agilent 7890B gas chromatograph coupled to an Agilent 5977B MSD system (Agilent Technologies Inc., Santa Clara, CA, USA), and an HP-5MS fused-silica capillary column (30 m × 0.25 mm × 0.25 μm, Agilent J & W Scientific, Folsom, CA, USA) was utilized to separate the derivatives. For LC–MS, an ACQUITY UPLC I-Class system (Waters Corporation, Milford, MA, USA) coupled with a VION IMS QTOF Mass spectrometer (Waters Corporation, Milford, USA) was used to analyze the metabolic profiling in both ESI positive and ESI negative ion modes. An ACQUITY UPLC BEH C18 column (1.7 μm, 2.1 × 100 mm) was employed in both positive and negative modes.

All raw data were imported into MS-DIAL software and Progenesis QI V2.3 software (Nonlinear, Dynamics, Newcastle, UK) for further identification processing. The missing values in the raw data were filled by using half of the minimum value. The identification of metabolites from GC–MS was based on the LUG database, while the detection of compounds by LC–MS was performed based on the Human Metabolome Database (HMDB), Metlin, PMDB, and self-built databases.

### 2.3. Statistical Analysis

Principal component analysis (PCA), orthogonal partial least-squares-discriminant analysis (OPLS-DA), and partial least-squares-discriminant analysis (PLS-DA) were performed using the R package to assess the overall distribution among the samples and evaluate the stability of the analysis process. Variable importance of projection (VIP) values obtained from the OPLS-DA were used to select metabolites for further analysis. Metabolites with a VIP value greater than 1 were subjected to Student’s t-test to determine the significance of the difference in their levels between the groups. ROC curve analysis was conducted, and the area under the curve (AUC) was calculated to assess the discriminatory abilities of the identified differential metabolites. Enriched metabolic pathways were identified using the Kyoto Encyclopedia of Genes and Genomes (KEGG) (http://www.genome.jp/kegg/) and MetaboAnalyst (http://www.metaboanalyst.ca/ accessed on 1 January 2020) [15,16]. 

All normally distributed variables are presented as the mean along with standard deviation (SD). Pearson’s correlation analysis was performed to investigate the associations between clinical parameters and the levels of differential metabolites. Statistical significance was set at a *p*-value of less than 0.05.

## 3. Results

**Figure 1:**
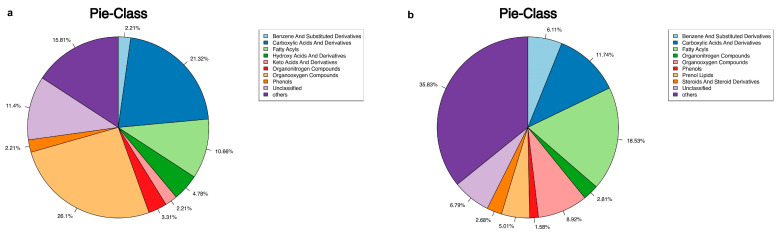
Pie charts of the main classes of metabolites identified by GC−MS (**a**) and LC−MS (**b**).

**Figure 2:**
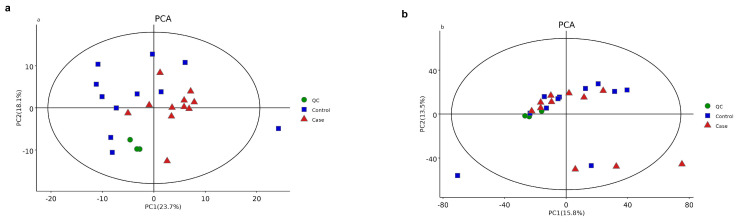
Principal component analysis (PCA) model showed significant differences between the CNV and control groups: (**a**) PCA model of GC−MS analysis; (**b**) PCA model of LC−MS analysis. Red dots in the figure represent CNV cases (n = 11); blue spots represent controls (n = 11); green spots represent quality control (n = 3).

**Figure 3:**
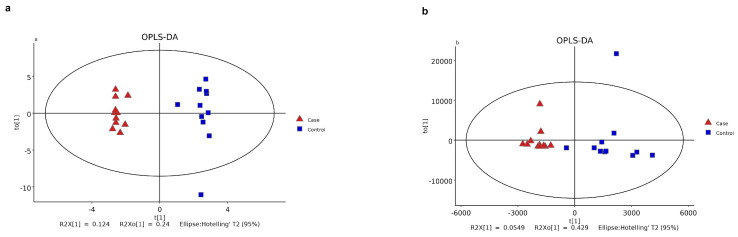
Orthogonal partial least-squares-discriminant analysis (OPLS−DA) model showed a significant difference between the CNV and control group: (**a**) OPLS-DA model of GC−MS analysis; (**b**) OPLS−DA model of LC−MS analysis. Red dots in the figure represent CNV cases (n = 11); blue spots represent controls (n = 11); green spots represent quality control (n = 3).

**Figure 4:**
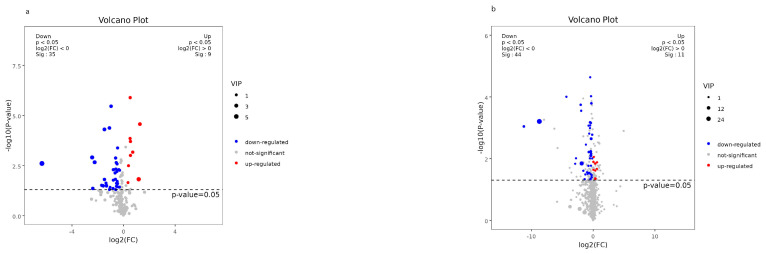
Volcano plots showing differential metabolites between groups from GC−MS (**a**) and LC−MS (**b**) analysis. Upregulated and downregulated metabolites are in red and blue, respectively. Nonsignificant metabolites are represented by gray dots.

**Figure 5:**
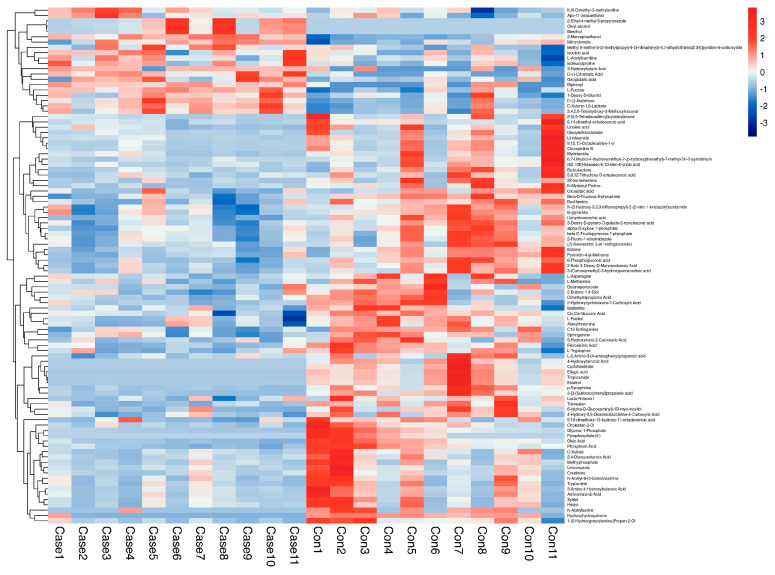
Heatmap showing relative peak areas of dysregulated metabolites in aqueous humor.

**Figure 6:**
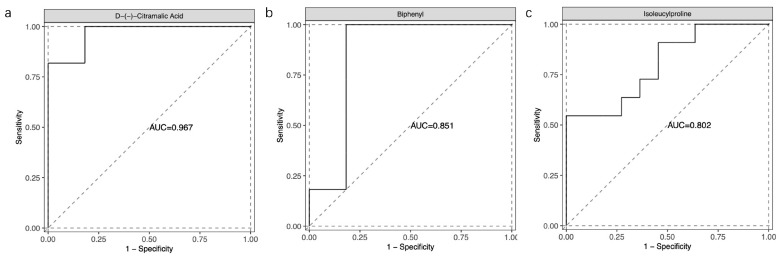
ROC curve of the three selected molecules.

Demographic characteristics of the patients, including age, sex, SER, and AL, were strictly matched between the CNV and control groups. The clinical features and fundus status of the patients are presented in Table 1. Among the CNV patients, three had retinal cystoid edema, and six had subretinal fluid (SRF). All values of differential metabolites between the two groups are provided in Supplementary Table S1. A total of 272 metabolites were detected in AH samples from both groups using GC–MS, while 1457 were identified using LC–MS (detailed in Figure 1a,b). PCA demonstrated distinct clustering of the quality control samples, indicating the validity of our analysis process (see Figure 2a,b). OPLS-DA score plots (Figure 3a,b) revealed a clear separation between the CNV and control groups. We successfully identified 97 differential metabolites as potential biomarkers for myopic CNV, based on a VIP value greater than 1 and a significance level of *p* < 0.05. To account for multiple testing, we applied the False Discovery Rate (FDR) method, and the corrected *p*-values are provided in the supplementary materials (Supplemental Table S1). Among these metabolites, 20 showed higher levels, while 77 showed lower levels in CNV patients compared to those in controls (see Figure 4a,b). Notably, the levels of estetrol (10 out of 11) and ellagic acid were found to be under the detectable limit in the CNV group, while oleyl alcohol, menthol, and 2-Ethyl-4-methyl-5-propyloxazole in the control group also exhibited levels below the detectable limit. For these cases, the missing values were filled with 0.006121 during the data analysis (Supplemental Table S1). K-medians clustering analysis further demonstrated the discriminatory capacity of these differential metabolites (see Figure 5). By applying a fold change (FC) threshold of greater than 1.5 or less than 0.667, six molecules, namely D-citramalic acid, biphenyl, isoleucylproline, menthol, 2-ethyl-4-methyl-5-propyloxazole, and oleyl alcohol, stood out due to their AUC values ranging from 0.801 to 1. However, it is worth noting that the levels of menthol, 2-ethyl-4-methyl-5-propyloxazole, and oleyl alcohol were found to be under the detectable limit in the CNV group, requiring further validation through additional investigations. Consequently, we selected three molecules (D-citramalic acid, biphenyl, and isoleucylproline) as potential disease-specific biomarkers [17,18] (see Table 2 and Figure 6). Then, the correlations among the clinical features and the levels of the three potential disease-specific biomarkers mentioned above were investigated by Pearson’s correlation analysis. The levels of the three potential disease-specific biomarkers showed no association with patient age, AL, or SER, but showed a strong association with the occurrence of SRF, with a *p*-value < 0.05. Additionally, the AH levels of isoleucylproline and D-citramalic acid were positively correlated with retinal cystoid edema.

**Figure 7:**
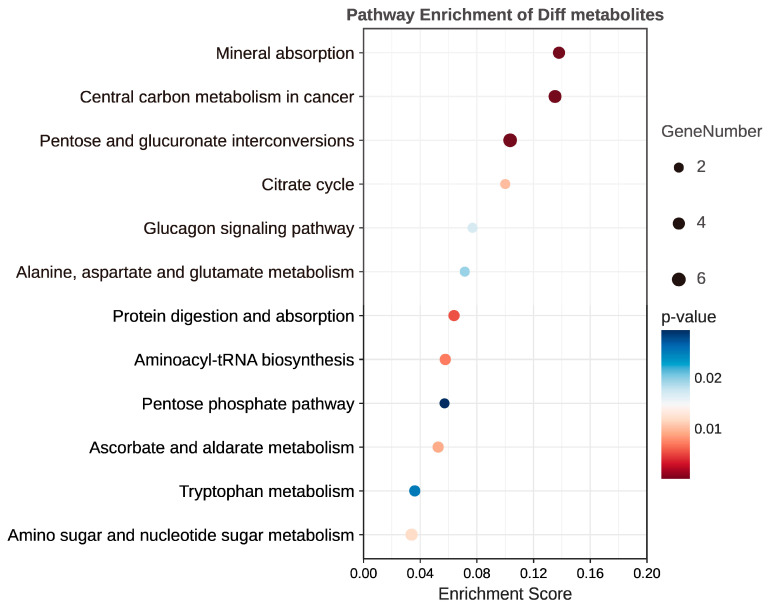
Metabolite pathway analysis showed the 12 most enriched metabolic pathways between the PM-related CNV and PM control groups. In this plot, “GeneNumber” refers to the number of genes associated with each metabolic pathway. The Enrichment Score represents the degree of enrichment for a specific pathway and is calculated based on statistical analysis, taking into account the relative abundance of metabolites and their association with the pathway. The higher the Enrichment Score, the more significantly enriched the pathway is in the PM-CNV group compared to the PM control group.

Metabolic pathway enrichment analysis identified 12 significantly enriched pathways. Among these pathways, five were related to carbohydrate metabolism, including pentose and glucuronate interconversions, citrate cycle (TCA cycle), amino sugar and nucleotide sugar metabolism, ascorbate and aldarate metabolism, and the pentose phosphate pathway. Two pathways were associated with amino acid metabolism, namely tryptophan metabolism and alanine, aspartate, and glutamate metabolism. The remaining five enriched pathways included central carbon metabolism in cancer, mineral absorption, the glucagon signaling pathway, protein digestion and absorption, and aminoacyl-tRNA biosynthesis (see Figure 7).

## 4. Discussion

Metabolomics has emerged as a promising research approach for the qualitative and quantitative analysis of metabolites in vivo. Over the past few decades, metabolomics has found successful applications in various fields of ophthalmology [8,9]. However, while there has been a growing number of untargeted metabolomic studies comparing metabolic differences between myopia and cataract controls, the number of myopic CNV-related metabolomics studies remains limited [19]. In addition, it is worth noting that the majority of published metabolomics studies related to myopia have primarily concentrated on analyzing plasma samples [19,20]. To the best of our knowledge, no previous studies have utilized AH as the sample of interest or employed PM patients as controls to explore the metabolomic characteristics of PM patients with CNV.

Different metabolite separation methods and statistical approaches allow for distinct classes of metabolite quantification [9]. In this study, we employed both liquid chromatography–mass spectrometry (LC–MS) and gas chromatography–mass spectrometry (GC–MS) to enhance the coverage of the metabolome. Using this approach, we identified 97 differential metabolites between the case and control groups, indicating metabolic and biomacromolecular disturbances during CNV formation. Among these, 77 molecules exhibited lower levels, while 20 showed higher levels in the CNV group compared to those in the controls. In two prior metabolomics studies related to high myopia using AH as samples, 29 and 21 differential compounds between high myopia and cataract controls were identified [12,13]. However, few of these findings are consistent with our results, which may be attributed to differences in the types of study participants and control groups. For instance, a study by Lian et al. reported higher concentrations of creatine and proline in the AH of PM patients compared to those in cataract controls, which was contrary to our findings [21]. Furthermore, it is important to highlight the findings of Liu et al., who reported upregulated expression of oleic acid in the serum of PM patients with CNV compared to that in cataract controls [19]. In contrast, our study revealed downregulated expression of oleic acid in the AH of PM-CNV patients compared to that in PM controls. This discrepancy can be attributed to several factors, including variations in age (38.4 ± 6.3 years vs. 38.5 ± 7.1 years in our study, 65.83 ± 11.94 years vs. 55.32 ± 14.49 years in the study by Liu et al.), differences in control groups (young PM patients in our study, patients with cataract in the study by Liu et al.), variances in biological samples (AH in our study, serum in the study by Liu et al.), and disparities in the fundus status of the included patients.

Numerous published studies have implicated the involvement of oxidative stress and inflammatory processes in the progression of PM and the development of PM-related complications [22,23]. The majority of molecules detected at decreased levels in the CNV group in our study have been reported to possess varying degrees of anti-inflammatory and antioxidant functions. In vivo, fatty acids demonstrate anti-inflammatory properties [24]. In our investigation, eight out of nine identified fatty acids and conjugates exhibited reduced levels in the CNV groups. Sphingolipids, including Clavepictine B and C16 sphinganine, recognized as important regulators of inflammation, angiogenesis, proliferation, migration, apoptosis, and fibrosis processes, displayed decreased levels in CNV patients [25]. Creatinine, which may act as an osmolyte safeguarding cells against hypertonic stress, as well as certain antibiotic and immunosuppressive molecules, showed reduced levels in the CNV group [26,27,28]. Significantly lower levels of L-fucitol, a compound with antibacterial properties, as well as p-synephrine and 9,12,15-octadecatrien-1-ol, known for their ability to suppress inflammatory responses, were observed in the CNV group compared to the controls [29]. The levels of three amines, namely oleoylethanolamide (OEA), 1-(2-hydroxypropylamino) propan-2-ol, and sphinganine, were found to be decreased in the CNV groups. OEA, in addition to its free radical scavenging properties and involvement in immune responses, has been reported to possess anti-inflammatory properties in vivo [30]. Furthermore, multiple studies have indicated the potential antioxidant properties of bucillamine, L-methionine, and ellagic acid (EA), all of which exhibited significantly lower levels in the CNV groups [31,32].

It is worth noting that our study revealed lower levels of several neuroprotective factors in the CNV group when compared to those of the controls. Our findings revealed deregulation of ectoine levels in patients with CNV. Ectoine, a natural protective factor, plays a crucial role in shielding proteins and biological membranes from damage caused by extreme environmental conditions or inflammatory cascades [33]. Dehydroascorbic acid (DHA) and cycloheximide, well-known neuroprotective factors, were also found to have reduced levels in the AH of CNV patients. Simultaneously, the AH of CNV patients exhibited significantly higher levels of toxic substances such as 2-mercaptoethanol, biphenyl, and oleyl alcohol. Among the molecules with increased levels in CNV patients, 2-mercaptoethanol, biphenyl, and oleyl alcohol have been found to be more toxic than ethanol to all tissues [34]. In comparison to PM controls, where oleyl alcohol levels were below the detectable limit, its concentration in CNV patients exhibited a wide range. However, whether it could serve as a biomarker of CNV, and the reasons for its absence in the controls, require further investigation. 1-Deoxy-D-glucitol could inhibit the activity of phosphofructokinase and hexokinases, thereby interfering with glucose metabolism. Our results demonstrated lower levels of 1-Deoxy-D-glucitol in the CNV group compared to that in the control group. L-fucose is typically expressed at very low levels in mammals under normal conditions. Serum and urine L-fucose levels have been utilized as disease biomarkers for cancer and gastric ulcers [35,36]. Whether the increased L-fucose level in AH could serve as a disease-specific biomarker for CNV formation requires further validation. In contrast to the other metabolites with increased levels, L-acetylcarnitine possesses distinctive neuroprotective properties, and oxoglutaric acid can inhibit the activity of ATP synthase and TOR6, extending the lifespan of adult C. elegans [37]. Further investigation is needed to ascertain whether these two metabolites can play a neuroprotective role during CNV formation. Consequently, the specific physiological and pathological mechanisms associated with these molecules in CNV formation warrant further study. D-citramalic acid, also known as 2-methylmalic acid, has been detected in the urine of patients with gut dysbiosis and autistic features, indicating its potential value in medical diagnoses [38,39]. However, further research is necessary to investigate the underlying reasons for its upregulation in PM-related CNV and to explore its diagnostic potential for CNV.

The concentration of menthol was undetectable in the PM control, while its expression level in CNV exhibited a wide concentration range. Menthol is a recognized inflammatory mediator that regulates TRP channels and can generate and release inflammatory mediators [40]. However, the precise biological function of menthol remains a subject of debate. Emerging evidence suggests that menthol may exert a protective role in various animal models by enhancing antioxidant enzyme activity, reducing reactive oxygen species generation, and modulating the production of TNF-α and interleukins [41]. Nevertheless, further investigation is warranted to elucidate its anti-inflammatory function in human AH. Increased isoleucylproline levels in AH were found to be associated with cystoid retinal edema and the occurrence of subretinal fluid (SRF) in our study. Isoleucylproline, a dipeptide comprising isoleucine and proline, can be hydrolyzed to generate active proline. However, proline exhibited significantly lower levels in CNV patients in our study (detail in Supplementary Table S1). Proline can interact with TRP channels to participate in the lipid signaling system, mediate metabolism between retinal pigment epithelium (RPE) cells and the retina, serve as an alternative energy source during stress or hypoxia, control mitochondrial function to exert antioxidant effects, and maintain redox homeostasis [42]. Hence, we speculate that the decrease in proline levels in AH may be attributable to the blocked hydrolysis of isoleucylproline, which could be a critical step in CNV formation. To date, only a limited number of articles have been published on 2-ethyl-4-methyl-5-propyloxazole. In our study, 2-ethyl-4-methyl-5-propyloxazole was found to be undetectable in the control group, while its expression levels in CNV patients exhibited a wide range of concentrations, indicating the need for further investigation to elucidate its physiological function. Isocitrate and ketoglutarate, intermediate products of the tricarboxylic acid (TCA) cycle, exhibit significant upregulation in patients with CNVs, suggesting the potential involvement of the TCA cycle in CNV formation.

Previous studies have identified the TCA cycle and sphingolipid metabolism as the most significantly enriched pathways in the serum of myopia patients using metabolomic analysis [9]. Metabolic analysis of AH from myopia patients revealed that alanine, aspartate, and glutamate metabolism, as well as arginine biosynthesis, were the most significantly enriched metabolic pathways [9] The results of Lian et al.’s study showed that bile secretion and tryptophan metabolism were enriched based on analysis of the AH humor of PM patients compared to that of cataract controls [21]. In our study, tryptophan metabolism exhibited greater enrichment in CNV patients compared to that in controls, further supporting the potential influence of tryptophan metabolism on the development of severe myopia-related CNV complications. Additionally, the bile secretion pathway was identified in both groups in our study with no significant differences in the expression level. Using cataract controls, Liu et al. concluded that altered thiamine metabolism, arginine and proline metabolism, and purine metabolism, based on the analysis of serum from PM patients, may contribute to the development of CNV formation [19]. However, none of these pathways exhibited greater enrichment in the AH of CNV patients compared to that of PM controls in our study.

Carbohydrate metabolism, as a fundamental biochemical process, ensures a continuous supply of energy to living cells. In our study, five carbohydrate metabolism pathways were found to be significantly enriched, supporting the notion that glycometabolism and energy metabolism disorders may play a crucial role in CNV formation in PM patients. Intravitreal injections of anti-vascular endothelial growth factor (VEGF) agents are considered the primary treatment for macular neovascular disease. However, despite standardized anti-VEGF therapy, some patients still experience persistent fluid or recurrent exudation. Based on these findings, we hypothesized that simultaneous targeting of carbohydrate metabolism and VEGF-related hypoxic metabolism disorders could offer a promising therapeutic strategy.

To the best of our knowledge, our study is the first to conduct untargeted metabolomic analysis of AH samples to differentiate between PM patients with and without CNV. The CNV patients and controls were well-matched, eliminating confounding factors such as age, sex, and systemic disease. Furthermore, we employed a dual metabolomics analysis strategy to enhance metabolite detection coverage. However, the main limitation of our study was the small sample size, which may have affected the reliability of the results. To mitigate the influence of age-related factors (e.g., cataracts and systemic diseases) on metabolic results, we exclusively collected AH from CNV patients aged 25 to 50 years. Consequently, it was challenging to recruit age-, sex-, and AL-matched controls from PM patients undergoing ICL implantation at our hospital, as most of these patients are younger than 30 years. These selection criteria resulted in a small cohort of patients meeting the research criteria. Another potential limitation of our study is its cross-sectional design and utilization of untargeted metabolic analysis, which makes it challenging to differentiate between causative and consequential differences in metabolomic profiles. Consequently, the specific metabolites and metabolic pathways that are altered during the formation of choroidal neovascularization (CNV) may not be definitively identified through our approach. Thus, conducting a larger cohort study with combined untargeted and targeted metabolic analysis is necessary to validate the findings of our study. Additionally, many of the identified metabolic features in our findings remain unexplored but are of clinical significance for in-depth investigations of myopic CNV. These unexplored metabolite features deserve attention in future studies.

## 5. Conclusions

In conclusion, our study utilized GC–MS and LC–MS-based metabolomic analysis to identify several disease-specific biomarkers for diagnosing PM-related CNV. Our findings indicate a significant role in CNV formation, suggesting the potential involvement of disturbed carbohydrate metabolism. However, larger studies are needed to validate and corroborate these observations fully.

## Figures and Tables

**Table 1:** Patients’ characteristics.

Characteristics	PM with CNV	PM Controls	*p* *
Age, y	38.4 ± 6.3	38.5 ± 7.1	0.976
Male/Female,	4/7	3/8	0.666
Axial Length, mm	28.0 ± 1.6	27.5 ± 1.1	0.406
SER, D	−11.2 ± 2.9	−9.5 ± 3.4	0.232
Grades of myopic degeneration (*n*) #	C0,1; C1,1; C2,3; C3,6	C0,4; C1,5; C2,1; C3,1	0.004
Retinal cystoid edema (*n*)	3	0	0.002
Subretinal fluid (*n*)	6	0	0.067

PM, pathological myopia; CNV, choroidal neovascularization; SER, spherical equivalent refractive error; D, diopters; *, *t*-test. #, grade of myopic degeneration: C0, no macular lesions; C1, tessellated fundus; C2, diffuse chorioretinal atrophy; C3, patchy chorioretinal atrophy; C4, macular atrophy.

**Table 2:** Sensitivity, specificity, and area under the curve values of potential disease-specific biomarkers.

Metabolite	VIP	FC Values	AUC	Specificity	Sensitivity	Cut-Off (uM)	95% CI
D-Citramalic Acid	2.62	2.4	0.967	1	0.818	0.0006	0.91–1.00
Biphenyl	1.80	1.6	0.851	0.818	1	0.0004	0.65–1.00
Isoleucylproline	1.05	1.5	0.801	1	0.545	11,810.81	0.62–0.99

VIP, variable importance of projection; FC, fold change; AUC, area under the curve.

## Data Availability

The data supporting the results of the current study can be found within the article and the supplementary material.

## Supplementary Materials

The following supporting information can be downloaded at: https://www.mdpi.com/article/10.3390/metabo13080900/s1, Supplementary Table S1.
